# Molecular Basis of Primary Aldosteronism and Adrenal Cushing Syndrome

**DOI:** 10.1210/jendso/bvaa075

**Published:** 2020-06-29

**Authors:** Patricia Vaduva, Fideline Bonnet, Jérôme Bertherat

**Affiliations:** 1 Reference Center for Rare Adrenal Diseases, Department of Endocrinology, Assistance Publique Hôpitaux de Paris, Hôpital Cochin, Paris, France; 2 Institut Cochin, INSERM U1016, CNRS UMR8104, Paris University, Paris, France; 3 Hormonal Biology Laboratory, Assistance Publique Hôpitaux de Paris, Hôpital Cochin, Paris, France

**Keywords:** Cushing syndrome, primary aldosteronism, cAMP, CTNNB1

## Abstract

This review reports the main molecular alterations leading to development of benign cortisol- and/or aldosterone-secreting adrenal tumors. Causes of adrenal Cushing syndrome can be divided in 2 groups: multiple bilateral tumors or adenomas secreting cortisol. Bilateral causes are mainly primary pigmented nodular adrenocortical disease, most of the time due to *PRKAR1A* germline-inactivating mutations, and primary bilateral macronodular adrenal hyperplasia that can be caused in some rare syndromic cases by germline-inactivating mutations of *MEN1*, *APC*, and *FH* and of *ARMC5* in isolated forms. *PRKACA* somatic-activating mutations are the main alterations in unilateral cortisol-producing adenomas. In primary hyperaldosteronism (PA), familial forms were identified in 1% to 5% of cases: familial hyperaldosteronism type I (FH-I) due to a chimeric *CYP11B1/CYP11B2* hybrid gene, FH-II due to *CLCN-2* germline mutations, FH-III due to *KCNJ5* germline mutations, FH-IV due to *CACNA1H* germline mutations and PA, and seizures and neurological abnormalities syndrome due to *CACNA1D* germline mutations. Several somatic mutations have been found in aldosterone-producing adenomas in *KCNJ5*, *ATP1A1*, *ATP2B3*, *CACNA1D*, and *CTNNB1* genes.

In addition to these genetic alterations, genome-wide approaches identified several new alterations in transcriptome, methylome, and miRnome studies, highlighting new pathways involved in steroid dysregulation.

Adrenocortical tumors are quite frequent; most of them are incidentally discovered (incidentalomas) ranging from 1% to 7% in the general population, with their frequency increasing with age. Unilateral benign adrenocortical tumors have various patterns of steroid secretion, from clinically inactive to overt Cushing syndrome or aldosterone secretion [1]. New mass spectrometry techniques revealed that most of the tumors considered as clinically inactive are in fact producing an excess of some steroids [2, 3]. In adrenal incidentalomas, cortisol excess leading to Cushing syndrome is seen in about 15% of cases, and aldosterone excess from Conn adenomas is seen in around 3% of cases [4-7].

Bilateral adrenocortical tumors are less common. Cortisol-secreting tumors can be roughly divided in 2 entities depending on the size of the nodules (micro- or macronodular adrenocortical hyperplasia).

Different mechanisms of tumorigenesis are involved in the growth of these adrenal tumors, with alterations in various signaling pathways. There are several points of interest in understanding their alterations:

- Understanding the mechanism of steroid secretion
- Exploring mechanisms of tumorigenesis, not only in these benign tumors but also in malignant tumors, using the same metabolic pathways
- Public health importance as these tumors are common and often associated with cardiovascular morbidity [8]
- Better patient care as classifying tumors with similar pathogenesis leads to an easier choice for medical or surgical treatment and adequate genetic counseling
- Easier diagnosis, thanks to specific markers detected by noninvasive techniques as micro ribonucleic acids (miRNAs) [9-11] or circulation tumor DNA [12, 13].

## 1. Adrenal Cushing Syndrome

**Figure 1.**
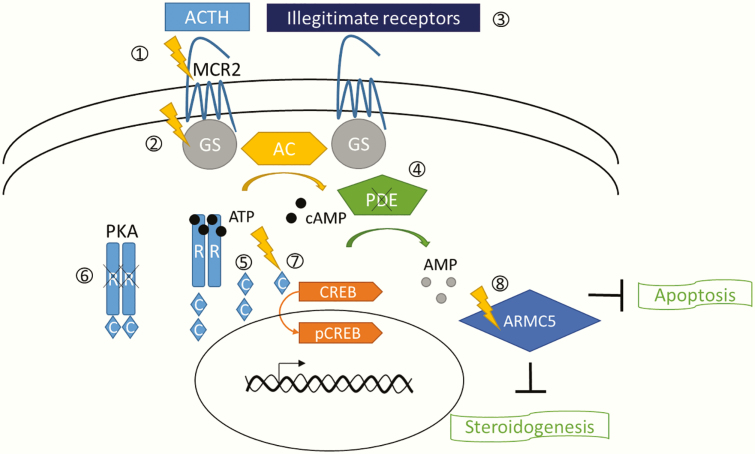
Signaling pathways and molecular alterations in adrenal Cushing syndrome. Physiologically, adrenocorticotropic hormone (ACTH) binds to a G protein–coupled receptor, the melanocortin receptor (MC2R), resulting in Gs protein activation. This in turn activates adenylate cyclase (AC) leading to cyclic adenosine monophosphate (cAMP) production. Four cAMP molecules bind to the protein kinase A (PKA) regulatory subunits dimer, which allows the release and activation of the 2 catalytic subunits of PKA. The free catalytic subunits will phosphorylate the transcription factor CREB (cAMP response element-binding protein), stimulating the transcription of several cAMP-dependant genes. Phosphodiesterases (PDE) involved in cAMP degradation are negative regulators of this pathway. Here is the list of molecular alterations disrupting cAMP/PKA signaling pathway which have been reported in the different etiologies of adrenal Cushing syndrome (mentioned in italics). 1. Activating mutation of *MC2R (PBMAH)* 2. Activating mutation of *GNAS1* (*germline mutation in McCune-Albright syndrome and somatic mutation in unilateral cortisol-secreting adenoma*) 3. Illegitimate G protein–coupled receptors expression *(PBMAH)* 4. Phosphodiesterase (*PDE11A and/or PDE8B*)- inactivating mutations (*PPNAD and PBMAH)* 5. *PRKACA* duplication *(bilateral,adrenal hyperplasia/micronodular and macronodular forms)* 6. *PRKAR1A*-inactivating mutations or deletion (germline mutation and somatic second hit)(*PPNAD*) 7. Activating somatic mutation of *PRKACA (unilateral cortisol-secreting adenoma)* Apart from cAMP/PKA pathway: 8. *ARMC5* inactivation (germline mutation and somatic second hit) reducing steroidogenesisand adrenocortical cells apoptosis (*PBMAH*)

In normal adrenocortical cells, the pituitary hormone adrenocorticotropic hormone (ACTH) binds to its 7-transmembrane G protein–coupled receptor melanocortin 2 receptor (MC2R), resulting in G protein activation, then adenylyl cyclase activation, and finally cyclic adenosine monophosphate (cAMP) production. The binding of 4 cAMP molecules to a PKA (protein kinase A) regulatory subunits dimer allows the release and subsequent activation of the 2 catalytic subunits. These catalytic subunits will further phosphorylate several nuclear and cytoplasmic targets, including the transcription factor CREB (cAMP response element-binding protein), responsible for the stimulation of cAMP-dependent gene transcription. The negative regulators of this pathway are the phosphodiesterases, which are responsible for cAMP degradation. Constitutive activation of the cAMP/PKA pathway can lead to tumorigenesis and Cushing syndrome as ACTH stimulates both adrenocortical cell growth and cortisol synthesis (Fig. 1).

### A. Bilateral nodular hyperplasia

Bilateral adrenocortical tumors cover a spectrum of several entities. Micronodular adrenal hyperplasia (MiAH) is defined by multiples nodules, less than 1 cm in diameter, in each adrenal. The most common form of MiAH is the primary pigmented nodular adrenal disease (PPNAD). Macronodular hyperplasia is defined by nodules more than 1 cm diameter, the most common cause being the primary bilateral macronodular adrenal hyperplasia (PBMAH). The bilateral nature of these tumors suggests a possible genetic predisposition; several mutations have been identified in these tumors through candidate gene approaches.

**Figure 2.**
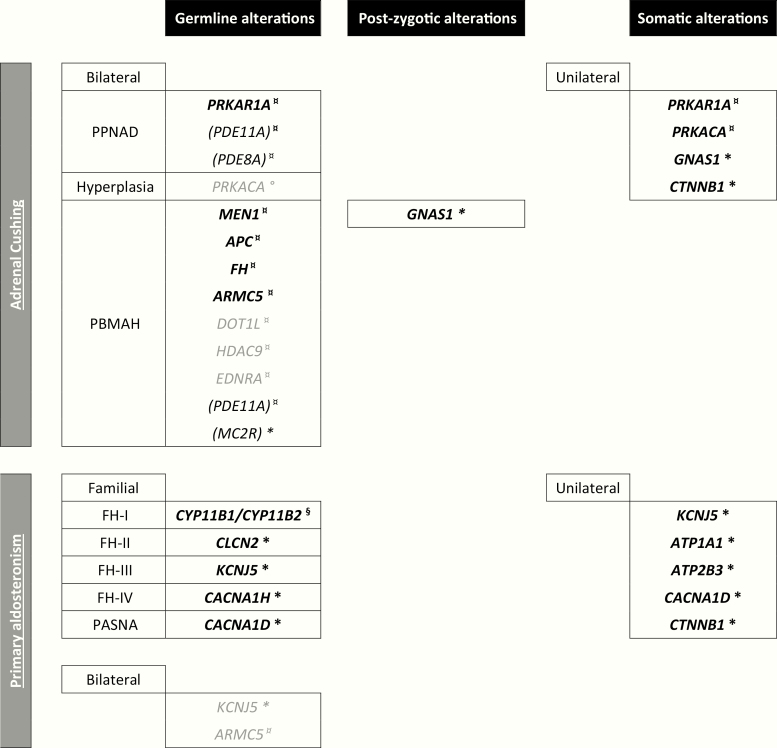
Genetic alterations in adrenal Cushing syndrome and primary aldosteronism. Type of genetic alterations: ^¤^ loss of function; * gain of function; ° duplication; ^§^ chimeric fusion gene. Gene function: **bold black** = certain causal gene; gray = causal gene to be confirmed; (brackets black) = causal/modifier gene. FH, familial hyperaldosteronism; PASNA, primary aldosteronism, seizures, and neurological abnormality; PBMAH, primary bilateral macronodular adrenal hyperplasia; PPNAD, primary pigmented nodular adrenal disease.

Initially, studies of families with segregation of several bilateral adrenocortical tumors identified the cAMP/PKA pathway as the first altered signaling pathway leading to tumorigenesis. Since these studies, other pathways have been identified as responsible of adrenal tumors (Fig. 2).

### PPNAD.

PPNAD is a rare cause of ACTH-independent Cushing syndrome, most often diagnosed in children and young adults and characterized by the presence of pigmented micro nodules (diameter less then 1 cm by definition but, in fact, often less than 3 mm) widespread in the cortex of both adrenals. PPNAD is the most frequent endocrine manifestation of the Carney complex (CNC), a tumor predisposition syndrome, reported in 26% to 60% of patients [14, 15], but it can also be isolated without tumors suggestive of CNC in 12% of the patients [14]. The clinical presentation is variable, with more or less marked hypercortisolism and sudden, insidious, or cyclic onset [16, 17].

CNC is familial in 70% of cases, with an autosomal dominant transmission. In the majority of cases, the disease is due to an inactivating mutation of the *PRKAR1A* gene, coding for the type I alpha regulatory subunit of PKA, located in 17q22-24. These mutations are found in 37% of cases with sporadic CNC and more than 70% of cases with familial CNC, with almost complete penetrance [14, 18, 19].

The first *PRKAR1A* mutations identified were frame shift mutations, leading to a premature stop codon [18]. The mutant messenger RNA was therefore unstable and degraded by nonsense-mediated messenger RNA decay. Often within the tumor deoxyribonucleic acid (DNA), a loss of heterozygosity (LOH) is observed, leading to loss of the wild-type allele in CNC tumors. The PKA regulatory subunit R1A protein is then lacking in tumor cells, resulting in the constitutive activation of cAMP/PKA pathway. *PRKAR1A* is considered as a tumor suppressor gene. No clear hotspot mutations were identified, and mutations are distributed all along the coding sequence and even sometimes in noncoding sequence (intronic mutations affecting splicing). However, 2 mutations were found with a higher prevalence: c.709-7del6 (intron 7) and c.491-492delTG (exon 5) [15]. The first one was mainly seen in isolated PPNAD, and the second was significantly associated with cardiac myxoma, lentigines, and thyroid tumors. The mutations escaping nonsense-mediated mRNA decay (20%) can give rise to the expression of an altered protein and have been suggested to be responsible for a more aggressive form of CNC.

For some cases of CNC, linkage studies revealed a second potential locus in 2p16 responsible for CNC. This region included the *POMC* gene and DNA-mismatch repair gene *MSH2*, but these 2 genes were further excluded [20]. The anomalies found at this locus were generally losses of heterozygosity and gains in the number of copies, suggesting the potential presence of an oncogene, which has not been determined to date [20, 21].

In addition, anomalies in the regulation of the catalytic subunits of PKA were reported. Triplication of the 1p31.1 chromosome region, including the *PRKACB* gene, were observed in a patient with CNC and having abnormal skin pigmentation, myxomas, and acromegaly but no PPNAD [22]. Duplication of the 19p region, including the *PRKACA* gene, was identified in patients with bilateral adrenal hyperplasia (micro nodular and macro nodular forms) and Cushing syndrome of various degrees [23].

Other actors of the cAMP/PKA pathway have been described as altered in patients with PPNAD. Three germline deleterious variants of *PDE11A* were identified in patients with MiAH/PPNAD without *PRKAR1A* mutation [24], suggesting a possible causative role of this gene. Moreover, single nucleotide variants of *PDE11A*, with decreased enzymatic activity in vitro [24], were found more often in patients with CNC due to *PRKAR1A* mutations that present with PPNAD than without PPNAD [25]. So *PDE11A* could also act as a modifier gene in patients with CNC. The single base substitution c.914A>C was identified in another phosphodiesterase gene*, PDE8A*, in a patient with PPNAD and early onset of Cushing syndrome [23]. Alterations of *PDE11A* and *PDE8B* were not specific of PPNAD and were further described in other types of adrenocortical tumors: PBMAH, adrenocortical adenomas (ACAs), nonsecreting adrenocortical adenoma and adrenocortical carcinomas (ACCs) [26-28].

In addition to the cAMP/PKA pathway, involvement of the Wnt/beta-catenin pathway was also reported in a study including 13 patients with PPNAD or sporadic cortisol-secreting adenomas (ACAs) with *PRKAR1A* somatic mutation. Beta-catenin accumulation was found in all the tumors studied and somatic mutations in *CTNNB1* and gene coding for beta-catenin were identified in some tumors [29]. This result was confirmed in another study which reported 2 *CTNNB1* somatic mutations in 18 patients with PPNAD [30].

### PBMAH.

PBMAH is usually defined by the presence of bilateral adrenal macronodules and cortisol autonomous secretion with low circulating ACTH, even if unilateral nodules with enlargement of the contralateral gland have been reported. The median age of diagnosis is around 50 years. The degree of cortisol autonomous secretion ranges from overt Cushing syndrome to almost hormonally inactive forms with minimal or subtle hormonal alterations.

Historically this disease was called ACTH-independent macronodular adrenal hyperplasia, but the name changed when it was found that in some forms of the disease, intra-adrenal ACTH synthesis was responsible for local stimulation of cortisol production [31].

In case of the rare syndromic forms of PBMAH (genetic tumor predisposition syndromes), multiple genetic alterations are known. Mutations of the *MEN 1* gene lead to type 1 multiple endocrine neoplasia, mutations of the *APC* gene lead to familial adenomatous polyposis and, and alterations of the *FH* gene lead to hereditary leiomyomatosis [32-35]. These genetic causes are, however, rarely found as isolated PBMAH is by far the most common form of the disease.

As in MiAH/PPNAD, genetic alterations of actors of the cAMP/PKA pathway were reported in PBMAH. *PDE11A* variants were found with a high prevalence in patients with PBMAH (24%-28%) [27, 36], some of them being associated with decreased enzymatic activity confirmed in vitro [36]. Activating mutations of the ACTH receptor (*MC2R*) gene are very rare but have been reported [37]. Post zygotic activating mutations of the *GNAS 1* gene, resulting in constitutive activation of the cAMP/PKA pathway and autonomous cortisol secretion, are causing McCune-Albright syndrome, with PBMAH [38, 39] in very young children.

In cases of isolated bilateral macronodular adrenal hyperplasia, the bilateral nature of the adrenal involvement and the existence of familial cases suggested the existence of a genetic cause for PBMAH. In the study of blood and tumor DNA samples of 33 patients with PBMAH who had undergone adrenal surgery, LOH was detected at the 16p locus by single nucleotide polymorphism array in 24% of the cases [40]. Whole genome sequencing of 5 paired tumor and leucocyte DNA samples identified *ARMC5* gene alterations, mapping to 16p11.2, as responsible for PBMAH. A first inactivating alteration was observed in the patients’ leukocyte DNA and a second event in somatic DNA extracted from adrenal nodules, suggesting a tumor suppressor role of the *ARMC5* gene. Inactivation of the second allele was due to LOH or point mutations, different from one nodule to another in the same patient. Other studies also confirmed the involvement of *ARMC5* in the pathophysiology of PBMAH in cohorts with sporadic or family cases [41-44]. The prevalence of *ARMC5*-damaging germline mutations in PBMAH with a sporadic presentation was further estimated from 21% to 26% in subsequent studies [41, 45]. Among patients with PBMAH, those having *ARMC5* mutations present with higher cortisol secretion levels, larger adrenals and a higher number of adrenocortical nodules, when compared with patients without *ARMC5* mutation [45, 46].

The precise function of *ARMC5* is yet unknown. It is a cytosolic protein, containing 7 armadillo domains, beta-catenin, and a Broad-Complex, Tramtrack and Bric a brac domain [47]. Functional studies showed that inactivation of *ARMC5* decreases expression of steroidogenesis enzymes and cortisol secretion in adrenal cells in vitro, but at the same time, expression of *ARMC5* mutant reduces apoptosis by comparison with the wild-type ARMC5 protein [40, 45]. This suggests that the increased number of adrenocortical cells explains the excess in cortisol secretion in patients with PBMAH, despite the reduced capacity of each cell [40]. It has been more recently shown that wild-type ARMC5 protein interacts with cullin 3 (*CUL3*). Missense variants of *ARMC5* losing the ability to interact with *CUL3* are not ubiquitinated and further degraded by the proteasome, and this could take part in cell cycle dysregulation [48].

Whole exome sequencing (WES) studies in patients with PBMAH reported alterations of other potential causal genes as *DOT1L* (coding for a histone H3 lysine methyl-transferase), *HDAC9* (coding for a histone deacetylase), and endothelin receptor type A (*EDNRA*) gene [49, 50].

Finally, PBMAH might be due to illegitimate membrane receptors on adrenocortical cells, cortisol secretion being secondary to nonphysiological stimuli. The stimulating ligands binding to G protein–coupled receptors are various: glucose-dependent insulinotropic peptide receptor (GIPR) responsible for food-dependent Cushing syndrome [51, 52], luteinizing hormone/human chorionic gonadotropin receptor responsible for Cushing syndrome during pregnancy and after menopause [53], and vasopressin, catecholamine, serotonin (5-hydroxytryptamine), angiotensin II, and glucagon receptors [54-60]. The prevalence of illegitimate membrane receptors in PBMAH is high, varying from 77% to 87% among studies [59, 60]. In vitro and animals studies for the GIPR showed the role of this ectopic expression on adrenal tumorigenesis and excessive cortisol secretion secondary to cAMP/PKA pathway activation [61, 62]. At present in PBMAH, no genetic alteration has been given to explain the mechanism of illegitimate membrane receptors despite whole genome approaches [63]. However, in unilateral adenoma with GIPR ectopic expression, gene rearrangement has been recently found at the *GIPR* locus [64].

### B. Unilateral adenomas

As in bilateral cortisol-secreting tumors, alterations of the cAMP/PKA pathway were described in unilateral cortisol-secreting adenomas. In 2014, these were identified by 4 independent teams, activating somatic mutations of the PKA catalytic alpha subunit (*PRKACA*) [23, 49, 65, 66]. Approximately 40% of cortisol-producing adenomas (CPAs) harbor *PRKACA* mutations, most of them presenting the hotspot mutation L206R [67]. In patients with CPAs carrying a somatic *PRKACA* mutation, the clinical phenotype was more severe than wild-type ones, and in fact, the mutations are only found in CPAs responsible for overt Cushing syndrome. An activating somatic mutation of the catalytic subunit beta of the PKA gene (*PRKACB*) has also been reported in CPAs as responsible for overt Cushing syndrome, but this alteration is apparently rare [68].

Somatic alterations of *PRKAR1A* were also described in CPAs, with LOH in the *PRKAR1A* locus 17q found in 7 of the 29 studied adenomas and somatic-inactivating mutations in 3 CPAs responsible for overt Cushing syndrome [69]. A *PRKAR1A* somatic mutation was also more recently described in a WES study, including 39 CPAs [49]. Somatic-activating mutations of *GNAS 1*, the gene coding for Gs protein alpha subunit, were also reported in a few rare cases of CPAs [65, 70, 71].

Activation of cAMP/PKA signaling leads to different pathway alterations in CPAs. In *GNAS 1*-mutated tumors, an overexpression of extracellular matrix receptor interaction and focal adhesion pathways was observed, while in *PRKAR1A*-mutated tumors, genes related to the Wnt signaling pathway are overexpressed [72]. Activation of the Wnt/beta-catenin pathway was also reported in around 40% of adrenocortical adenomas without any somatic mutation of *PRKAR1A*, most of them being explained by the occurrence of a somatic-activating mutation of the beta-catenin gene (*CTNNB1*) [73]. Activating mutations in *CTNNB1* are more frequent in nonsecreting adrenal adenomas [74, 75]. This suggests that the consequences Wnt/beta-catenin activation on cortisol dysregulation differs from the one of cAMP/PKA signaling activation.

### C. Omics alterations in adrenal Cushing syndrome

In chromosome alteration studies comparing ACAs to adrenocortical carcinomas, the first group shows a smaller proportion of copy number alteration and loss of heterozygosity [76, 77]. The 9q34 region, including the steroidogenic factor 1 locus, is commonly gained in ACA [77].

Transcriptome studies showed various genes differentially expressed between ACAs and ACCs, with upregulation of steroidogenic genes in ACAs, compared with ACCs [78] and overexpression of insulin-like growth factor 2 and insulin-like growth factor 2-related genes in ACCs. A distinct gene expression profile in patients with overt Cushing syndrome versus subclinical Cushing syndrome and nonfunctional tumors confirmed the activation of the cAMP/PKA pathway and overexpression of steroidogenic genes [79]. Lampron et al showed that 723 differentially expressed genes are identified in GIP-dependent tumors, including perilipin, overt expression of 13 G-protein coupled receptors and potential involvement of Rho-GTPases [80].

Transcriptome, as well as methylome and microRNA (miRNA) studies are limited at present in ACA but show interesting results in aldosterone-producing adenomas.

## 2. Benign Adrenal Tumors Associated with Primary Hyperaldosteronism

**Figure 3.**
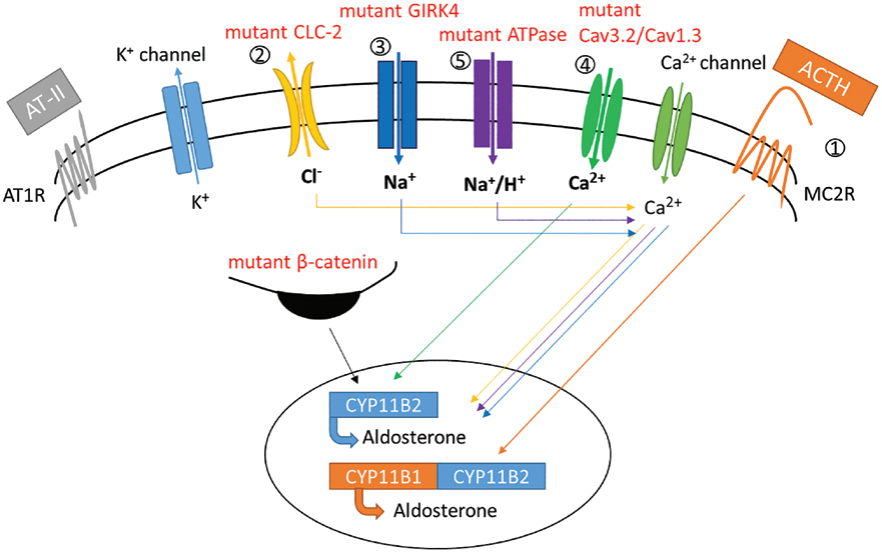
Molecular alterations in primary hyperaldosteronism. Physiologically, aldosterone production is stimulated by angiotensin II (AT-II) and extracellular potassium. Indeed, both stimuli induce glomerula cells membrane depolarization, leading to opening of voltage-gated calcium channels. Calcium influx triggers a phosphorylation cascade inducing aldosterone synthase expression and aldosterone production. Here is the list of the molecular alterations responsible for the different forms of primary hyperaldosteronism 1. Expression of the hybrid gene *CYP11B1/CYP11B2 (germline alteration in FH-I)* making aldosterone production dependent on ACTH regulation and thus responsible for “glucocorticoid-remediable hyperaldosteronism” 2. Gain-of-function mutations in *CLCN-2* gene *(germline mutation in FH-II)*, responsible for constitutive membrane depolarization of glomerulosa cells by increased chloride efflux. 3. Mutations in *KCNJ5* gene affecting particularly ion selectivity of the K+ channel thus responsible for an increased sodium influx leading to membrane depolarization *(germline mutation in FH-III and somatic mutations in APAs).* 4. Mutations in CACNA1H *(germline mutation in FH-IV)* and *CACNA1D* genes (germline mutation in PASNA syndrome and somatic mutations in APAs) responsible for increased calcium membrane permeability. 5. Somatic mutations in *ATP1A1* and *ATP2B3* genes responsible for increased permeability for Na+ or H+ resulting in membrane depolarization of cell membrane by due to variants (*in APAs*) 6. Somatic mutations in *CTNNB1* gene responsible of increased *CYP11B2* expression (*in APAs*). ACTH: adrenocorticotropic hormone AT1R: angiotensin receptor; MC2R: melanocortin receptor.

The 2 most common causes of primary hyperaldosteronism (PA) are aldosterone-producing adenomas (APAs), also called Conn adenomas, or bilateral adrenal hyperplasia (BAH). APAs are usually unilateral, small in size (1-2 cm), and diagnosed in patients aged 40 to 50 years [81-87]. BAH accounts for 60% to 70% of patients with PA [88]. Aldosterone synthesis is tightly regulated by the renin-angiotensin system and extracellular potassium concentration. Angiotensin II (Ang II), binding to its Ang II type 1 receptors, stimulates the inositol triphosphate signaling pathway, inducing a calcium (Ca^2+^) release from the endoplasmic reticulum. Stimulation by potassium and Ang II also result in zona glomerulosa cell membrane depolarization, responsible for the opening of voltage-dependent Ca^2+^ channels. Both signals contribute to increase intracellular Ca^2+^ concentration, triggering a phosphorylation cascade, leading to increased transcription of enzymes responsible for aldosterone synthesis (Fig. 3). While the majority of cases of PA are sporadic, 1% to 5% of cases are inherited familial forms, transmitted as autosomal dominant traits. Four different forms have been described based on the underlying genetic defects [89-91]. At the same time, recurrent somatic mutations in several genes have been identified in 88% of APAs [92] (Fig. 2).

### A. Genes associated with familial hyperaldosteronism

#### Familial hyperaldosteronism type I.

 Familial hyperaldosteronism type I (FH-I), also reported as glucocorticoid-remediable aldosteronism, is an autosomal dominant disease, described for the time in 1966 in a father and his son suffering from hypertension due to PA [93]. The particularity of their phenotype relied on aldosterone suppression and therefore hypertension resolution with dexamethasone treatment. FH-I is usually due to bilateral hyperplasia or, in rare cases, adrenal nodules and is associated with a significant production of the hybrid steroids 18-hydroxycortisol and 18-oxocortisol. Clinical and biochemical characteristics are variable, even within the same family. In the adult hypertensive population, FH-I accounts for 0.5% to 1% of PA, occurring in the same proportion in men and women. The molecular origin of FH-I was elucidated by Lifton et al in 1992 who identified a chimeric *CYP11B1/CYP11B2* hybrid gene in patients with FH-I [94]. These 2 genes are highly homologous and localized in tandem on the chromosome 8q21-8q22, *CYP11B1* coding for 11-beta-hydroxylase, the enzyme responsible for the last steps of cortisol synthesis and *CYP11B2* coding for aldosterone synthase, the enzyme responsible for the last steps of aldosterone synthesis. The hybrid gene includes the promoter region of *CYP11B1* and a large part of the *CYP11B2* coding sequence, making aldosterone production dependent on ACTH regulation. Patients with FH-I are at increased cardiovascular risk, even for normotensive patients [95], with an increased number of cerebrovascular events at a young age [96].

#### Familial hyperaldosteronism type II.

 Familial hyperaldosteronism type II (FH-II), was first described by Gordon et al as a second form of autosomal dominant form of PA, not remediable with glucocorticoids and not due to the presence of the chimeric CYP11B1/CYP11B2 gene [97]. The prevalence of FH-II ranges from 1.2% to 6% in the adult population of patients with PA [88]. APAs and BAH have been reported with a high phenotypic variability, even within the same family [98-101]. FH-II is clinically and biochemically indistinguishable from sporadic forms of PA and is only diagnosed on the basis of 2 or more affected family members.

Linkage analysis found an association between FH-II and the chromosomal region 7p22, but no mutations were found in different candidate genes located in this region [102]. In 2018, 2 independent teams identified several gain-of-function mutations in the *CLCN2* gene, coding for the CLC2 chloride channel in patients with FH-II and early onset of PA [89, 90]. Mutations were located in different domains of the protein and could therefore explain the phenotypic heterogeneity. It was the first time that implication of a chloride channel was shown in the regulation of aldosterone production. A recent study also found 1 somatic mutation in *CLCN2*, after screening 80 apparently sporadic APAs, in a male patient with PA, which was cured after surgery of its small adenoma [103].

#### Familial hyperaldosteronism type III.

 Familial hyperaldosteronism type III (FH-III) was first described in 2008 by Geller et al in a father and 2 daughters with early-onset, severe resistant arterial hypertension and hypokalemia [104]. Their phenotype was particularly severe, associating marked hyperaldosteronism with very high levels of the hybrid steroids 18-oxocortisol and 18-hydroxycortisol but no suppression of aldosterone production by dexamethasone treatment and massive BAH. Bilateral adrenalectomy was necessary to control blood pressure. Recently, WES identified a germline mutation in the *KCNJ5* gene (p.Thr158Ala) in a patient with FH-III, unraveling the genetic origin of this syndrome [105]. *KCNJ5* is located on chromosome 11q24.3, and coding for the G protein-activated inward rectifier potassium channel GIRK4. This mutation affects channel ion selectivity for potassium (K^+^), leading to sodium (Na^+^) influx into the zona glomerulosa cell, cell membrane depolarization, activation of voltage-dependent Ca^2+^ channels, increase in intracellular Ca^2+^ concentration, and stimulation of steroidogenesis enzyme synthesis resulting in aldosterone synthesis.

Different germline *KCNJ5* mutations were further reported in families with FH-III. Patients carrying the germline mutations p.Gly151Arg, p.Thr158Ala, and p.Ile157Ser all presented a severe phenotype of PA, whereas affected members from 3 families with FH-III carrying the p.Gly151Glu mutation and from 1family carrying the p.Tyr152Cys mutation exhibited a milder phenotype similar to FH-II [88]. However, like for the other forms of familial hyperaldosteronism, a strict genotype-phenotype correlation is difficult to establish, and a certain degree of phenotypic variability exists.

#### Familial hyperaldosteronism type IV.

 In familial hyperaldosteronism type IV, T-type voltage calcium channels (Cav 3) are activated at a higher negative membrane potential and display voltage-dependent inactivation. The alpha-1 subunit is the main transmembrane and pore-forming portion of Cav3.2 channels and is encoded by *CACNA1H* gene located on chromosome 16p13.3 [106]. WES in blood samples from 40 patients with very early-onset hypertension identified a hotspot de novo mutation in *CACNA1H* p.M1549V in 5 cases [107]. Four germline mutations of the same gene were identified later in patients with diverse phenotypic presentation of PA [108]. A whole patch clamp study in HEK293T cells transfected with the mutant *CACNA1H* showed slower inactivation and longer opening in the channel, resulting in higher Ca^2+^influx [109].

#### PA, seizures, and neurological abnormalities syndrome.

*CACNA1D* mutations have been described in 2 children with PA, seizures, and neurological abnormalities. This gene, located on chromosome 3p14.3, encodes the alpha-1 subunit of L-type voltage Ca^2+^ channels (Cav1) [106]. These mutations induce a gain of function, with channel opening at a lower voltage, leading to excessive aldosterone production by increasing intracellular calcium levels. Exome sequencing in 100 patients with early-onset of PA identified 2 patients with germline de novo mutations in *CACNA1D* p.G403R and p.I770M [110]. The phenotype was very severe in both patients. The first patient, carrying a p.G403R mutation, presented with hypertension since birth, resulting in biventricular hypertrophy associated with ventricular septal defect and pulmonary hypertension. The second patient, harboring a pI770M mutation, was diagnosed with hypertension at 5 years old, while he presented central nervous system attempt (spastic quadriplegia and seizures) since birth.

### B. Somatic mutations in APAs

#### KCNJ5.

 The first gene whose somatic alteration was reported in APAs was *KCNJ5*, identified by WES of samples from patients with sporadic forms of APAs [81]. Two hotspot mutations were reported, p.G151R and p.L168R, respectively, localized in the highly conserved glycine-tyrosine-glycine motif of the selective filter and in the second transmembrane domain of *KCNJ5*. They both abolish K^+^ selectivity of the channel. The consequences of this alteration were further confirmed in other studies. *KCNJ5* mutations were thus identified in 180 (38%) of 474 APA samples collected from the European Network for the Study of Adrenal Tumor, the 2 hotspot mutations being largely predominant with respective prevalence of 63% for p.G151R and 36% for p.L168R [111]. The *KCNJ5* mutation prevalence in APAs was found to be higher in some specific populations, particularly in the Asian population [83], confirmed by a meta-analysis on 1636 patients, showing a prevalence of *KCNJ5* mutations of 43%, and up to 77% in populations from East Asia. APAs harboring mutations in *KCNJ5* were more frequent in women and young patients and were associated with larger tumors and higher plasma aldosterone concentrations [112].

#### Adenosine triphosphatases.

 Adenosine triphosphatase (ATPase) somatic mutations have also been reported in APAs. Na^+^/K^+^-ATPases are composed of alpha and beta subunits, with the alpha subunit including the Na^+^/K^+^ and ATP-binding sites and the beta subunit directing the alpha subunit to the plasma membrane. Na^+^/K^+^-ATPases transport 3 Na^+^ ions in exchange for 2 K^+^ ions, using the driving force of ATP hydrolysis and generating an electrochemical gradient across the membrane that facilitates ion cellular uptake. At least 4 alpha subunit isoforms have been described. *ATP1A1* codes for the alpha-1 subunit of Na^+^/K^+^-ATPase, located on chromosome 1p13.1 [106]. Three different somatic alterations of *ATP1A1* (2 substitutions, p.L104R and p.V332G and 1 deletion, p.F100_L104) were identified in 16 of 328 APAs (6.8%) by Beuschlein [113]. Expression in an adrenal cell line showed that ATPase activity of mutant proteins was severely impaired, with decreased Na^+^ and K^+^ binding, inducing high-level membrane depolarization, opening of voltage-gated calcium channels, and enhanced aldosterone production [113]. Thirteen different mutations were reported so far, with a global prevalence of 5% to 8% in patients with APAs among the studies [85, 106].

*ATP2B3*, located on chromosome Xq38, encodes the plasma membrane calcium transporter Ca^2+^-ATPase 3, including 2 calcium- and 1 calmodulin-binding sites. Ca^2+^-ATPases are in an autoinhibitory state under physiological Ca^2+^ intracellular concentration. Ca^2+^ transport in exchange to 1 H^+^ cation, using driving force of ATP hydrolysis, can be activated by calmodulin binding. In-frame deletion mutations of *ATP2B3* were reported in 1% to 1.5% of APAs [106]. These deletions were always located between the amino acids L424 and V429, leading to calcium-binding site distortion and inducing membrane depolarization in functional studies [113].

#### Calcium channels.

 WES of APAs identified somatic alterations in *CACNA1D*. These mutations were the same as those identified at the germline level, p.G403R and p.I770M [110]. Other somatic mutations along the coding region of *CACNA1D* were further identified [85], with a global prevalence of 3% to 11% in patients with APAs among the studies [106]. *CACNA1D* mutations were the most frequent ones in APAs from patients with African ancestry, up to 42% [114].

#### Beta-catenin.

 As in cortisol-producing adrenal tumors, beta-catenin accumulation in both nuclear and cytoplasmic compartments is very common in APAs with a prevalence of about 70% [115]. Nuclear beta-catenin can stimulate the expression of the transcription factor TCF/LEF, which further activates the transcription of transcription factors NURR1 and NURR7, finally activating the transcription of *CYP11B2* [115, 116]. Interestingly, in transgenic mice harboring beta-catenin inactivation specifically targeted in the adrenal cortex, hyperproliferation of zona glomerulosa cells and PA were observed in 10-month-old mice [117]. However, *CTNNB1* mutations were rare, found in approximately 3% of sporadic APAs, suggesting the Wnt/beta-catenin pathway activation through other mechanisms [85, 110]. Somatic mutations of *CTNNB1* have been associated to female gender and relatively large adenomas.

### C. Aldosterone-producing cell clusters

In the normal human adrenal gland, aldosterone-producing cell clusters (APCCs) have been recently observed, and it has been suggested that they autonomously produce aldosterone. APCCs are increased in patients with PA and negative computed tomography [118]. APCCs are characterized by a uniform expression of CYP11B2, in cell clusters and non-CYP11B1 expression and are composed of subcapsular zona glomerulosa-like cells and inner large zona fasciculata-like cells [119-121].

Approximately 35% of APCCs harbor mutations observed in APAs, causing aldosterone overproduction—*CACNA1D* mainly and *ATP1A1*, but no mutation in *KCNJ5* [122].

APCCs and APAs are differentiated by their size, cellular arrangements, and enzyme expression profile as APAs are composed of heterogeneous cell types expressing either CYP11B1 or CYP11B2. Recently described were some transitional structures, consisting of a subcapsular APCC-like structure and an inner micro-APA-like structure without a well-defined histological border, called possible APCC-to-APA transitional lesions and characterized by the presence of *KCNJ5* and *ATP1A1* mutations [121, 123]. Thus, some APAs could derive from APCCs with CACNA1D and ATP1A1 mutations or from possible APCC-to-APA transitional lesions, but the precise mechanism of these transitions has not been found yet.

### D. Molecular basis of bilateral hyperaldosteronism

Contrary to APAs, for which molecular determinants are well established, the molecular basis of bilateral hyperaldosteronism and bilateral adrenal cell proliferation are unknown. In 2011, it was shown that *KCNJ5* mutations were not only involved in driving aldosterone secretion but also in promoting cell proliferation [105]. Thus, a study carried out on 251 patients affected by sporadic bilateral hyperaldosteronism revealed 3 heterozygous missense germline mutations in *KCNJ5*, 2 of them resulting in membrane depolarization [124]. *ARMC5* germline variants, first described in PBMAH, were also reported in patients with PA. In a cohort of 56 patients with PA, some of them presenting with bilateral adrenal hyperplasia, almost 40% carried a genetic variant in the *ARMC5* gene, among which about one-fourth were predicted to be deleterious in in silico analysis [86]. However, all concerned patients were African American, and this association was not confirmed in a white cohort of 39 patients presenting with primary aldosteronism and bilateral adrenal hyperplasia. Eleven common variants, 5 rare variants and 2 unknown variants, were indeed identified in this cohort but none of them was predicted to alter protein function [125], so the role of *ARMC5* in PA needs further confirmation.

### E. Omics alterations in APAs

A recent study performed deep quantitative proteomic profiling on APAs and adjacent nontumoral adrenal tissue and showed 11 significatively upregulated proteins out of 5555. This upregulation concerned steroidogenic enzymes as HSD3B2, CYP21A2, and CYP11B2 and proteins involved in cholesterol uptake as a lipolysis-stimulated lipoprotein receptor [126]. Higher levels of proteins involved in N-glycosylation and enzymes involved in gamma-aminobutyric acid (GABA) degradation are seen in APAs. N-glycosylation affects the activity of steroidogenesis regulators such as MC2R (adrenocorticotropic hormone receptor), AT1R (Ang II receptor), lipoprotein receptors, and ion channels. GABAergic signaling mediates a steroidogenesis decrease in vivo, in rat adrenal cortex [127].

Moreover, the activity of the mammalian target of rapamycin pathway involved in cell proliferation, steroidogenesis, and immortalized adrenocortical cells is increased in APAs [128]. Inhibiting this pathway could then be a new perspective of treating patients with APAs.

In APAs, Bassett et al revealed upregulation of genes encoding transcription factors NURR1 and NGF1B that regulate CYP11B2 as well as SF-1 and DAX1 and that play a role in adrenal development and steroidogenesis [129, 130].

There are also some specific transcriptomic markers of APCCs. In addition to CYP11B2, genes like SLC35F1 (role in glucose transport), MC2R (role in aldosterone production), and PPP4R4 (role in phosphorylation) have higher transcript expression in APCCs compared with zona glomerulosa or fasciculata [122]. Thus, aldosterone synthesis in APCC, known to be independent of renin and angiotensin II stimulation, could be regulated, at least partially, by ACTH [122].

Based on differential gene expression, we are now able to distinguish APAs with *KCNJ5* somatic mutations from *ATP1A1, ATP2B3*, and wild-type tumors [131, 132].

Several microRNAs (small size, noncoding RNAs that promote translational repression) such as miR-24, miR-23b, miR-34a, miR-203, and miR-375 were identified in APAs and CPAs as involved in modulating steroid biosynthesis and secretion by different mechanisms such as modulation of *CYP11B1* and *CYP11B2* expression [133-136].

APAs are characterized by a global hypomethylation, compared with normal adrenal and nonfunctioning adenomas, correlating to upregulation of *CYP11B2* and genes involved in tumorigenesis [137, 138] as well as higher demethylation of G protein–coupled receptors and G protein–coupled receptor-related genes [139].

## 3. Concomitant Aldosterone and Cortisol Production

Several reports described conditions related to glucocorticoid excess: insulin-resistant type 2 diabetes mellitus, osteoporosis, depression, and anxiety in patients with primary aldosteronism [140, 141]. However, current guidelines for diagnosis of PA do not indicate hypercortisolism assessment [142].

In 2015, a study compared steroid hormone production in patients with aldosterone-producing adenomas to patients with bilateral hyperplasia and showed that this last group had higher urinary, peripheral, and adrenal venous concentrations of the hybrid steroids 18-oxocortisol and 18-hydroxycortisol [143]. Two years later, a spectrometry-based analysis of 24-hour urine steroid metabolome showed that patients with PA had significantly increased cortisol and glucocorticoid metabolite excretion when compared with healthy controls, inactive adrenal adenomas, and mild subclinical adrenal cortisol excess, correlated with several parameters indicative of adverse metabolic risk [144]. Among patients with PA, those presenting with cortisol co-secretion, seem to have more sever left ventricular hypertrophy, as an additional impact on cardiac remodelling, compared with patient with aldosterone secretion only. Moreover, adrenalectomy has a better efficiency left ventricular mass index, than mineralocorticoid receptor therapy [145].

In the study by Arlt et al, the glucocorticoid excretion was significantly associated with intratumoral *CYP11B1* expression, required for the synthesis of glucocorticoids and 11beta-hydroxyandrostenedione, explaining also the normal or increased androgen output of these tumors [144].

Moreover, it has been shown that a distinct steroid signature can predict APA genotype in adrenal venous and peripheral plasma; for example, a 7-steroid fingerprint in peripheral plasma correctly classified 92% of the APAs according to the genotype [146]. High plasma levels of 18-hydroxycortisol and 18-oxocortisol have been identified in patients with APAs and were predictive of *KCNJ5* mutations. Furthermore, based on matrix-assisted laser desorption/ionization technology, it was shown that 137 metabolites were significantly different in patients with *KCNJ5* and *CACNA1D* mutations [147]. An increased intratumoral content of 18-oxocortisol and 18-hydroxycortisol was found in *KCNJ5*-mutated APAs. The 18-oxocortisol tumoral content and CYP11B1 expression levels (inversely correlated) were associated with outcome (higher probability of complete clinical success after surgery), independent of clinical parameters. Concordantly, Arnesen et al showed that *KCNJ5* somatic mutations were associated with a better surgical outcome in 28 APAs, which was confirmed recently by Vilela et al on 100 patients with unilateral PA, where somatic *KCNJ5* mutation was an independent predictor of hypertension remission after adrenalectomy [148, 149]. *ATP1A1*-mutated APAs had higher CYP11B2 staining intensities.

## 4. Conclusion

Molecular basis of cortisol-secreting adenomas is currently well defined, even if genetic explanation is still missing for some cases. Alterations of the cAMP/PKA pathway can explain the occurrence of a significant proportion of the tumors responsible for overt Cushing syndrome. Other pathways such as the Wnt/beta-catenin pathway or the still-to-be explained ARMC5 signaling pathway can also play a role in cortisol-secreting tumors that are often less active in terms of cortisol secretion. Major advances in determination of molecular mechanism leading to sporadic and familial primary aldosteronism have been made in the last years. Novel familial forms have been characterized. Somatic mutations driving aldosterone overproduction have been found in around 60% of sporadic APAs. Recent studies showed that some adenomas were producing concomitantly aldosterone and cortisol in excess.

## Abbreviations

ACA: adrenocortical adenoma
ACC: adrenocortical carcinoma
ACTH: adrenocorticotropic hormone
Ang II: angiotensin II
APA: aldosterone-producing adenoma
APCC: aldosterone-producing cell clusters
ATPase: adenosine triphosphatase
BAH: bilateral adrenal hyperplasia
Ca2+: calcium
CAMP: cyclic adenosine monophosphate
Cav: calcium channel
CNC: Carney complex
CPA: cortisol-producing adenoma
CUL3: cullin 3
DNA: deoxyribonucleic acid
FH: familial hyperaldosteronism
GABA: gamma-aminobutyric acid
GIPR: glucose-dependent insulinotropic peptide receptor
K+: potassium
LOH: loss of heterozygosity
MC2R: melanocortin 2 receptor
MiAH: micronodular adrenal hyperplasia
miRNA: microRNA
Na+: sodium
PA: primary hyperaldosteronism
PBMAH: primary bilateral macronodular adrenal hyperplasia
PKA: protein kinase A
PPNAD: primary pigmented nodular adrenal disease
RNA: ribonucleic acid
WES: whole exome sequencing

## Additional Information

***Disclosure Summary:*** There are no disclosures to declare. Both authors have submitted the ICMJE form for Disclosure of Potential Conflicts of Interest. Conflicts that the editors consider relevant to the content of the manuscript have been disclosed.

***Data Availability:*** Data sharing is not applicable to this article as no datasets were generated or analyzed during the current study.
